# A cell marker‐based clustering strategy (cmCluster) for precise cell type identification of scRNA‐seq data

**DOI:** 10.15302/J-QB-022-0311

**Published:** 2023-06-01

**Authors:** Yuwei Huang, Huidan Chang, Xiaoyi Chen, Jiayue Meng, Mengyao Han, Tao Huang, Liyun Yuan, Guoqing Zhang

**Affiliations:** ^1^ CAS Key Laboratory of Computational Biology Bio‐Med Big Data Center Shanghai Institute of Nutrition and Health University of Chinese Academy of Sciences Chinese Academy of Science Shanghai 200031 China; ^2^ Ningbo Institute of Life and Health Industry University of Chinese Academy of Sciences Ningbo 315000 China

**Keywords:** single‐cell RNA‐seq, clustering, cell markers, novel cell types

## Abstract

**Background:**

The precise and efficient analysis of single‐cell transcriptome data provides powerful support for studying the diversity of cell functions at the single‐cell level. The most important and challenging steps are cell clustering and recognition of cell populations. While the precision of clustering and annotation are considered separately in most current studies, it is worth attempting to develop an extensive and flexible strategy to balance clustering accuracy and biological explanation comprehensively.

**Methods:**

The cell marker‐based clustering strategy (cmCluster), which is a modified Louvain clustering method, aims to search the optimal clusters through genetic algorithm (GA) and grid search based on the cell type annotation results.

**Results:**

By applying cmCluster on a set of single‐cell transcriptome data, the results showed that it was beneficial for the recognition of cell populations and explanation of biological function even on the occasion of incomplete cell type information or multiple data resources. In addition, cmCluster also produced clear boundaries and appropriate subtypes with potential marker genes. The relevant code is available in GitHub website (huangyuwei301/cmCluster).

**Conclusions:**

We speculate that cmCluster provides researchers effective screening strategies to improve the accuracy of subsequent biological analysis, reduce artificial bias, and facilitate the comparison and analysis of multiple studies.

## Abstract 2

**Author summary:** The importance and challenge of clustering method for scRNA‐seq data is that recent methods introduce difficulty and bias in the identification of cell types and cell function explanation. We proposed a cell marker‐based clustering strategy (cmCluster) to determine accurate clusters by introducing a knowledge benchmark during the fine adjustment of clustering. cmCluster helped to obtain finer cell populations for single‐cell transcriptome data with both known and unknown cell type labels. And these populations will be suitable to identify cell types or acquire features especially for massive scRNA‐seq data with complex or potential novel cell types.

## INTRODUCTION

Single‐cell RNA sequencing (scRNA‐Seq) technology not only investigates the breathtaking functional diversity at the single‐cell level [1, 2, 3, 4, 5] but also provides rich and multi‐dimension cell data to explore the new types of cells and their biological function in certain sample [6, 7, 8, 9, 10, 11]. Therefore, as one of the most important steps in the analysis of scRNA‐seq data, clustering is the basis for recognition of cell populations and explanation of biological processes [12, 13, 14, 15, 16].

Current studies provide massive clustering methods that produce initial clusters for further cell type identification. One of the most widely used clustering algorithms is the community‐detection‐based Louvain algorithm, along with shared‐nearest‐neighbor graphs, including Distributed Stochastic Neighbor Embedding (t‐SNE) [17] and Uniform Manifold Approximation and Projection (UMAP) [18, 19, 20] to provide data‐driven, consistent and unbiased clusters [12,14] by Seurat [21] or Scanpy [22]. Besides, SC3 (single‐cell consensus clustering) [23] and Wagner tried to find clustering consensus from different clustering runs [24], which indicate that more precise clusters can be obtained through tuning parameters [25]. Even though multiple methods provide clusters with similar mathematical features, it is still difficult to match the clusters to the biological populations [12,26]. Not just those cells from different conditions are mislabeled as the same cell type, the rare cell types are also hard to recognize. Thus, scReclassify [27] proposes a semi‐supervised learning framework to effectively correct the mislabeled cells from the perspective of annotation. And RaceID identifies rare cell type through searching outliers while SIMLR searches rare cell type by custom distance measurement [28,29]. Furthermore, Jackson uses experiment cell markers for fine adjustment to obtain a more‐granular distinction of cell types [30]. These results indicated that biological information is helpful for recognition of cell populations during clustering and annotation [31]. However, there is not an extensive and flexible strategy to balance clustering accuracy and biological explanation during these steps.

**Figure 1:**
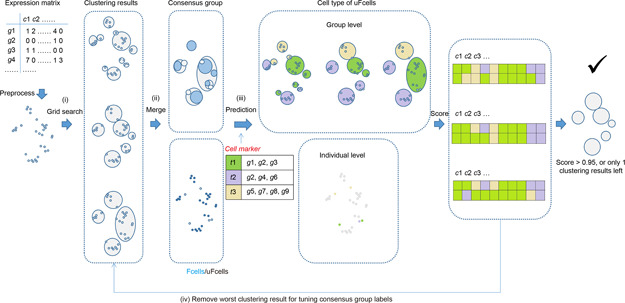
**The pipeline for cmCluster.** Overall workflow for cmCluster including (i) initial clustering with grid search; (ii) clustering consensus and Fcells/uFcells detection (Fcells were represented by blue solid points and uFcells were hollow ones); (iii) evaluate clustering results with agreement score for two groups of predicted cell types of uFcells; (iv) tuning consensus group labels and iteration. A little dot represents a single cell, while grey represents no predicted cell types and other colors (purple, green, yellow) represent different cell types. Each cycle represents a given cluster. Here, *g, c* and *t* represent gene, cell and cell type, respectively.

We therefore propose cell marker‐based strategy which integrated cell type annotation results during the iterative clustering to search the optimal clustering results by shifting the component and label of high variable cells in genetic algorithm (GA) within the range of grid search as describe in Fig.1. The accuracy of cell type label is used as the benchmark of GA iteration. This strategy is suitable for finding more precise clusters and more reliable sub‐clusters in both individual study and multiple parallel studies for massive scRNA‐seq datasets.

## RESULTS

### Parameter contribution of clustering and cell type prediction

**Figure 2:**
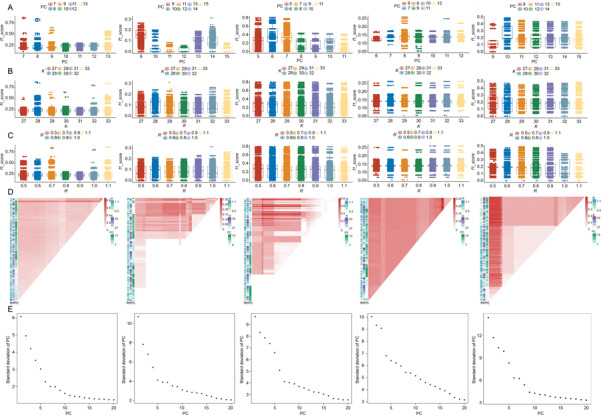
**Distribution of parameters for cmCluster iteration.** (A) Boxplot of f1‐score grouped by PC. (B) Boxplot of *f1*‐score grouped by K. (C) Boxplot of *f1*‐score grouped by R. (D) Heatmap of f1‐score for all parameter combinations during iteration of cmCluster. (E) Elbow plot of datasets.

Different combination of parameters led to diverse clustering results. Here, *f1‐score* described the consistency of uFcells between cell type prediction in individual and group expression level. During the iteration of cmCluster with different set of parameters, *f1‐score* varied in different clustering results as shown in Fig.2.

Principal component (PC) was more important in clustering than K‐nearest neighbors ( *K*) and Resolution ( *R*) because the *f1‐score* of the same PC were similar even though *K* and *R* showed great difference ( Fig.2) and only the median value of the *f1‐score* for different PC varied (Δ>0.06). In addition, the value of PC with highest median *f1‐score* was exactly the information saturation point in elbow plot in Fig.2, which indicated that an appropriate PC benefited clustering.

Furthermore, cmCluster was convenient to compare the *f1‐score* during iteration as shown in Fig.2. Here we took the top five parameter combinations to select the most suitable combination of parameters. The parameter combination with highest *f1‐score* or average *f1‐score* was selected (Supplementary Table S5). The decline of the *f1‐score* in some iteration was mainly due to the inconsistency of cell populations.

### Improvement on clustering results

The boundary and outlier of clusters selected by cmCluster were calculated to evaluate the precision of clustering and the effectiveness of biological annotation as shown in Materials and methods. For CALLR and SC3, clustering results were deficiency as a large amount of cells increased the memory and time sharply.

**Figure 3:**
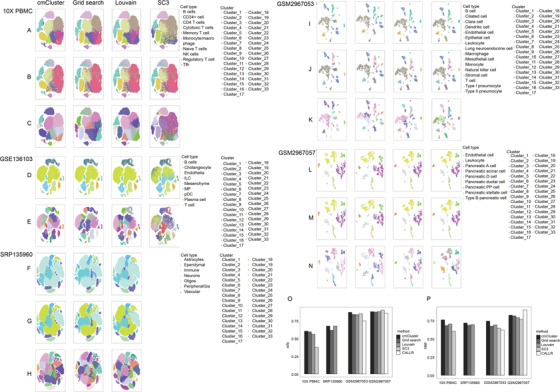
**Comparison of cluster and cell types among cmCluster and other methods, and the ARI/NMI for predicted cell types in all datasets.** t‐SNE plot of the 10X PBMC dataset with predicted labels (A), true labels (B), and clustering labels (C), respectively. t‐SNE plot of the GSE136103 dataset with predicted labels (D) and clustering labels (E). t‐SNE plot of the SRP135960 dataset with predicted labels (F), true labels (G), and clustering labels (H), respectively. t‐SNE plot of the GSM2967053 dataset with predicted labels (I), true labels (J), and cluster labels (K), respectively. t‐SNE plot of the GSM2967057 dataset with predicted labels (L), true labels (M), and clustering labels (N), respectively.

The clustering results of cmCluster were more precise than other methods in all datasets with clearer boundaries and less outliers as shown in Fig.3 and Supplementary Fig. S1. The median ratio of outliers for cmCluster was lowest in all datasets among all method (0.05) while SC3 provided massive clusters with highest median ratio of outliers (0.25) as shown in Supplementary Fig. S1. And the mean purity of clusters for cmCluster was highest among all methods except in datasets GSM2967053 as shown in Supplementary Fig. S2. In GSM2967053, cmCluster provided mean purity that only 0.03 less than SC3 while the number of cells in each cluster was twice than that of SC3. Furthermore, the clusters that differed in a comparison of cmCluster and other methods were checked carefully.

For datasets with known cell types like 10X PBMC datasets, Cluster 13 was not precise enough in Louvain, while the cells from the same cluster were split into two blocks in cmCluster as shown in Fig.3, respectively. In addition, the cluster which divided into two blocks in all of the other methods in Fig.3 was merged into cluster 1 after optimizing by cmCluster. Similarly, cluster 10 of datasets GSM2967053 in cmCluster was split in other methods when these cells should merge as endothelial cells. Besides, cluster 14 of cmCluster was confused with monocyte in other methods. Moreover, for datasets with unknown cell types such as datasets GSE136103 and SRP135960, similar conclusion can be drawn. In conclusion, not only the precision of cluster was increased by cmCluster, but also the compactness of cluster was increased.

### Benefit on cell type identification with standard labels

The biological performance of optimized clusters by cmCluster were checked in datasets (10X PBMC, SRP135960, GSM2967053 and GSM2967057). The ARI (adjust rand index as shown in Materials and methods) for each clustering method was calculated between the predicted and standard labels as shown in Fig.3. The ARI of cmCluster was higher than other methods in both datasets and no less than 0.6. At datasets SRP135960, the ARI of cmCluster was close to that of Louvain method as their parameters were very closed (PC, *K*, and *R* were 9, 31, and 0.7 in cmCluster, 8,30 and 0.8 in Louvain method, respectively) and all PC values were around the information saturation point as shown in elbow plot of Fig.2. For datasets GSM2967053 and GSM2967057, SC3 reached high ARI by dividing more clusters than cmCluster with lower NMI (normalized mutual information as shown in Materials and methods) as shown in Fig.3–P. And ARI of GSM2967057 between all methods were almost equivalent as the cell populations were significantly different between each other which made clusters similar between different methods. These indicated that cell type prediction was closer to true biological performance after optimization by cmCluster.

Next, the precision of predicted cell types was checked carefully. For datasets with known cell types, only cmCluster and grid search found all of ten cell types while other methods failed to identify different T cells in 10X PBMC datasets as shown in Fig.3. And cmCluster detected one more CD34^+^ cell type than grid search. For datasets GSM2967053 in Fig.3–K, SC3 sacrificed specificity to increase accuracy by giving more sub clusters and predicted more three cell types than cmCluster. However, the epithelial cells and B cells of SC3 were mislabeled while cmCluster not. Furthermore, only T cells and natural killer cells in cmCluster showed a clear gap. These indicated that the clusters optimized by cmCluster were more sensitive to cell type prediction. For datasets with unknown cell types such as GSE136103, only cmCluster with grid search successfully annotated cholangiocyte cells ( Fig.3–E). And in datasets SRP135960 in Fig.3–H, all methods found seven cell types and 75 percent astrocytes were annotated to neurons. This could be related to the insufficient of astrocytes cell markers. And we suggested users do not to conduct cell type prediction with cell types and their subtypes together.

In general, the clusters obtained by cmCluster performed better annotation results. Meanwhile, the integration of clustering and biological annotation showed the finest clusters compared with the application of any single factor.

### Effectiveness of cell subtype recognition

Because the clusters were more than cell types in studies, the subgroups were checked by detecting differential expressed genes (DEGs) to search for the most suitable biological characteristics.

**Figure 4:**
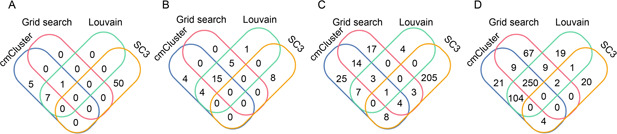
**Subtype detection in 10X PBMC datasets.** (A) Overlap of DEGs between subtypes of B cells in cmCluster, grid search, Louvain and SC3 groups, respectively. (B) Overlap of DEGs between subtypes of CD34 cells in cmCluster, grid search, Louvain and SC3 groups, respectively. (C) Overlap and expression level of DEGs between subtypes of Monocyte/Macrophage in cmCluster, grid search, Louvain and SC3 groups, respectively.

For known cell types, there were three main parts (B cells, CD34 cells and Monocyte/Macrophage) that were different after using cmCluster in 10X PBMC datasets, as shown in Fig.3–C. The DEGs between subtypes of B cells for cmCluster, grid search and Louvain shown a high degree of consistency while SC3 provided totally different DEGs that were not related to B cells in Fig.4. Besides, the four of five genes only detected by cmCluster such as *VPREB3, CD1C, GAPDH* and *IGJ* were report as gene markers of B cells and the rest one was related to immune function in database CellMarker. The DEGs between subtypes of CD34 cells for cmCluster, grid search and Louvain shared a common set of DEGs and cmCluster detected four distinct genes as shown in Fig.4. Similarly, only the DEGs of Monocyte/Macrophage provided by cmCluster shared most common genes with other methods in Fig.4.

For unknown cell types, the DEGs between subtypes of endothelial cells with novel cell type were compared between the group of cmCluster and other methods to evaluate the effectiveness of novel subtype identification. Both *ACKR1* and *VWA1* gene were only defined as DEGs after cmCluster, which indicated that novel *CD34* ^+^ *PLVAP* ^+^ *ACKR1* ^+^ and *CD34* ^+^ *PLVAP* ^+^ *VWA1* ^+^ endothelial cells were detected by cmCluster while none of the novel subtypes appeared in other methods ( Fig.4). In particular, the key gene *ACKR1* showed significantly different expression in cmCluster with a log‐fold change of 1.82.

In conclusion, these results indicated that cmCluster provided more precise clusters close to biological cell types. Furthermore, the precise division of existing cell types would be helpful for detecting novel cell types as the biological function between clusters varies.

## DISCUSSION

Most existing clustering methods only cluster cells based on the mathematical characteristics of single‐cell transcriptomes and introduce difficulty in the explanation of biological meaning. Therefore, this paper proposed cmCluster for large single‐cell transcriptome datasets to determine accurate clusters by introducing a knowledge benchmark during the fine adjustment of clustering.

The usage of a knowledge benchmark during clustering could improve the importance of biological features and retain the overall similarity of the expression matrix. The flexibility of method and the fineness of clustering make it convenient to adjust multiple parameters at once. The cmCluster results for the tested datasets showed that the proper parameter combination was located near the default value. We therefore suggest a slide window with a default value as the center and a window size of seven for the determination of the parameter range. Accurate clusters with biological interpretability increase the consistency of cell distribution in the same cell types and reduce the disturbance of noise cells to help reveal cell function.

A benchmark for knowledge of comprehensive cell markers may have a great impact on downstream analysis since cell type identification requires biological knowledge to identify clusters of certain cell types, especially for novel cells. cmCluster performs annotation twice for each single cell using the same marker genes to overcome such limitations and then compares the cell label and its group label. The common standard of cell type identification reduces the bias of gene markers by dividing similar cells into the same group, which is more robust for clustering.

Massive cells increase the consumption of both computational memory and the time needed for analysis sharply. cmCluster annotated only about 10% of the cells and took about 1 day for every dataset for the two test datasets including 40,000–70,000 cells. The balance between data size and resources should be considered.

Overall, cmCluster is a standard strategy that is easy to operate, and it can be refined to make up for artificial deviations. It is highly extensible for users to choose accurate clusters and identify cell types in massive scRNA‐seq data, and it is especially suitable for complex cell types or potential novel cell types.

## MATERIALS AND METHODS

### General concept of cmCluster

The cmCluster strategy, which is a modified Louvain clustering method, searched the optimal clusters through genetic algorithm (GA) and grid search based on the cell type annotation results. After matrix preprocessing (Supplementary File S1), the strategy was summarized by the following four steps as shown in Fig.1: (i) generate a set of initial clustering results through grid search under all combination of input parameter values; (ii) each cell was then given a consensus label based on the clustering results with minimum groups, and divided into Fcells (cells with fixed label in different clustering results) and uFcells (cells with unfixed label in different clustering results); (iii) predict cell types of uFcells by individual cell expression level or average group expression level, and evaluate the accuracy of each clustering result through calculating the agreement score of above predicted labels; (iv) tuning consensus group labels after removing the clustering results with lowest agreement score, and repeat step (ii) to (iv) until all clustering results with the agreement score over 0.95 or only one clustering result was left.

### Clustering consensus and Fcells/uFcells selection

We selected Louvain to test our strategy as this method was widely used in clustering of single cells. Louvain provided three main parameters to produce clusters including Principal Component (PC), K‐nearest neighbors ( *K*) and Resolution ( *R*) [21], and suggested users to try proper clusters by giving default values (PC near the knee point ( *kp*) of elbow plot, *K* and *R* were 30 and 0.8 in formula 1, respectively). Here, we produced initial clustering results (ICR) by grid search with a combination of these three parameters. Each parameter contained a slide window of the same width ( *W)* with a center of default values. The initial clustering results (ICR) is represented by I $CR=\left\{c|c=C_{PC}^{1}C_{K}^{1}C_{R}^{1}\right\}$ , and $card\left(ICR\right)=W^{3}$ .

(1) $$\begin{array}{rl} & PC=\left\{kp-\frac{W-1}{2},\cdots ,kp-1,kp,kp+1,\cdots ,kp+\frac{W-1}{2}\right\}\\ & K=\left\{30-\frac{W-1}{2},\cdots ,29,30,31,\cdots ,30+\frac{W-1}{2}\right\}\\ & R=\left\{0.8-0.1\times \frac{W-1}{2},\cdots ,0.7,0.8,0.9,\cdots ,0.8+0.1\times \frac{W-1}{2}\right\} \end{array}$$

All of the ICR was scanned to dete rmine the benchmark with the minimum groups, and each ICR was then relabeled according to the benchmark by the similarity of cell fraction, under the hypothesis that consensus group always shared same single cells. The expression level of some single cells may vary even though they belong to the same cell type due to the limitations of technique and instability of the cell state [27]. These cells that caused bias for clustering were defined as uFcells (unfixed‐label cells or noise cells). In contrast, cells that always showed in the same group were defined as Fcells.

### Gene marker selection and cell type prediction

In order to introduce biological characteristics into GA, gene marker was used to describe the feature of uFcells. The gene marker was defined as the high expressed gene specific in a cell type. The gene marker list was acquired from previous article, experiment or related database such as CellMarker [32]. As for the lack of gene markers for novel cell type, a set of experimental gene markers for the rest known cell types were strongly recommended. When the classification of known cell types was precise enough, a group of cells that was different from all of the know cell types was a potential reliable novel cell type.

Then the cell type of a uFcell will be predicted twice including average group expression level and individual cell expression level to overcome the uncertainty of prediction method itself. An expression matrix (${EXP}_{m\times n}$ with *m* genes and *n* cells) in step (ii) will be split into uFcell (noise cell) matrix (${NOISE}_{m\times n^{\prime }}$ with *m* genes and $n^{\prime }$ cells) and Fcell matrix (${AGREE}_{m\times (n-n^{\prime })}$ with *m* genes and $\left(n-n^{\prime }\right)$ cells) first. And the consensus group labels of each cell were defined as cell meta information set (MI) that each element contains all of the cells with the same group label.

(2) { EXPm×n=(expij)m×n NOISEm×n′=(nexpij)m×n′ AGREEm×(n−n′)=(aexpij)m×(n−n′)

**Figure 5:**
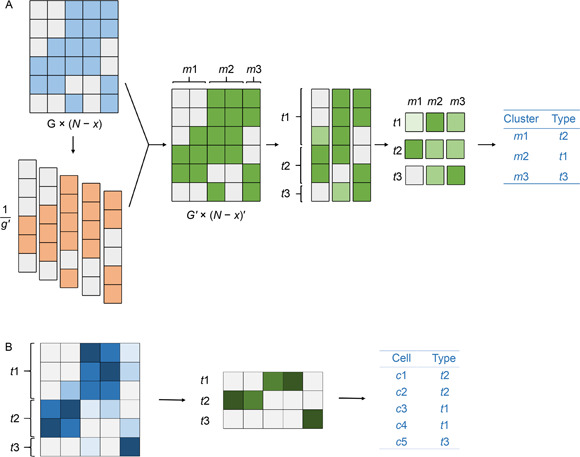
**Workflow for cell type prediction of noise cells in similar group level and single cell level.** (A) Cell type prediction of noise cells in group level would be done by evaluating the weighted expression level of marker genes of each Fcell that shares the same consensus group label of uFcells as described in matrix PSG where the light of color represents the range of expression. The expression of each gene would be weighted according to the whole expression level of all marker genes in a cell and scored by CPS to be integrated by cell types and consensus groups. The light of color in CPS represents the range of prediction score. Finally, each cluster would be tagged as the cell type with highest expression level. (B) Cell type prediction of uFcells in individual level would be done according to expression level of marker genes.

The cell type prediction of a uFcell in average group expression level were acquired by the predicted label of similar Fcells that share the consensus group label. Cell type prediction of Fcell matrix were conducted as described in Fig.5. During this prediction, sub expression matrix of marker genes for Fcells (${SEXP}_{m^{\prime }\times (n-n^{\prime })}={\left({sexp}_{ij}\right)}_{m^{\prime }\times \left(n-n^{\prime }\right)}$ ) were weighted by detection rate ( *dr*) into weighted expression matrix (${WEXP}_{m^{\prime }\times (n-n^{\prime })}={\left({sexp}_{ij}\times {dr}_{ij}\right)}_{m^{\prime }\times \left(n-n^{\prime }\right)}={\left(w{exp}_{ij}\right)}_{m^{\prime }\times \left(n-n^{\prime }\right)}$ ) first. Here, *dr* was defined as the ratio of marker genes whose count was more than zero. Then a matrix of cell‐type‐predicted score (CPS) was calculated by the average of weighted expression matrix according to the consensus group labels of cells and the cell types of marker genes to define the probability of given cell types for all consensus groups. The predicted cell type for Fcells were determined by the maximum of CPS, and uFcells that shared the same consensus group label with these Fcells will obtain the same predicted cell type.

As for cell type prediction of uFcells in individual expression level, the posteriori probability of cell types with the expression level of marker genes was used. cmCluster identified the cell types using cellassign [33] to annotate uFcells for markers provided by the original data source or CellMarker database [32] (Supplementary Table S1).

### Evaluation of cell type prediction and tuning consensus group labels

In order to select the clustering results with better biological characteristics, the consistency between cell type prediction in average group expression level and individual cell expression level was taken as the standard during the iteration of parameter selection. The agreement score was defined by *f1‐score* [34] to describe this consistency.

(3)  f1_score=2∗precision∗recallprecision+recall precision=TPTP+FP recall=TPTP+FN

Cell type prediction in average group expression level was taken as the true label because Fcells showed similar expression pattern. *TP*, *FP*, *TN* and *FN* represented the true positive rate, false positive, true negative and false negative respectively. Only the clustering result with the lowest *f1‐score* were deleted in one iteration.

### Evaluation of clustering performance

We evaluated the cluster by classification accuracy and biological interpretability (purity, confusion matrix and the list of DEGs). The accuracy of classification will be quantified in two ways including the consistence between standard and predicted labels of cell types (adjust rand index (ARI)) and the concentration of clustering results (normalised mutual information (NMI) and the ratio of outliers beyond boundary) [35]. ARI and NMI can be calculated as below. Where $n_{ij}$ represented the number overlap cells between standard label *i* and predicted label *j*, $a_{i}=\sum_{i}n_{ij}$, $b_{j}=\sum_{j}n_{ij}$. P(i) and $P^{\prime }\left(j\right)$ represented the probability distribution function of *i* and *j* respectively, and P(i,j) represented joint probability distribution function of *i* and *j*.

(4) $$ARI=\frac{\sum_{ij}\left(\begin{array}{c} n_{ij}\\ 2 \end{array}\right)-\left[\sum_{i}\left(\begin{array}{c} a_{i}\\ 2 \end{array}\right)\sum_{j}\left(\begin{array}{c} b_{j}\\ 2 \end{array}\right)\right]/\left(\begin{array}{c} n\\ 2 \end{array}\right)}{\frac{1}{2}\left[\sum_{i}\left(\begin{array}{c} a_{i}\\ 2 \end{array}\right)+\sum_{j}\left(\begin{array}{c} b_{j}\\ 2 \end{array}\right)\right]-\left[\sum_{i}\left(\begin{array}{c} a_{i}\\ 2 \end{array}\right)\sum_{j}\left(\begin{array}{c} b_{j}\\ 2 \end{array}\right)\right]/\left(\begin{array}{c} n\\ 2 \end{array}\right)}$$

$$NMI\;=\frac{2\sum_{i}\sum_{j}P\left(i,j\right)log\left(\frac{P\left(i,j\right)}{P\left(i\right)P^{\prime }\left(j\right)}\right)}{-\sum_{i}P\left(i\right)log\left(P\left(i\right)\right)-\sum_{j}P^{\prime }\left(j\right)log\left(P^{\prime }\left(j\right)\right)}$$

Next, the boundary of the cluster was defined as the congregation of cells whose neighbors came from the other cluster. The outliers were the cells far away from the boundary when 90% of neighbor cells were from different cluster. The ratio of outliers in the boundary cells showed if the cluster was suitable enough to exclude potential misclassified cells. Therefore, the lower the ratio of outliers, the more accurate the clusters were.

Cluster purity was defined as the ratio of the true cell type within cluster to describe the consistency of cell population. And the average purity for all clusters was calculated as formula 5, where $n_{ij}$ represented the number of cells for true cell type *j* in cluster *i*, and $N_{i}$ represented the number of all cells in cluster *i* when *N* cells were divided into *m* clusters from *k* true cell types in total.

(5) $$average\_purity=\sum_{i=1,j=1}^{i=m,j=k}\frac{\max(n_{ij})}{N_{i}}$$

Finally, the confusion matrix [36] was used to check if the clustering results were accurate enough to gather same cell types together.

### Benchmarking

In order to evaluate the applicability and effectiveness of our strategy, public data were carefully selected as our standard based on the following criterias. (i) scRNA‐seq data with both known and unknown cell types were used to test the distribution of biological knowledge for intense adjustment of clustering and function description. (ii) Datasets with novel cell types were selected due to the challenge of clustering with incomplete or uncertain cell markers. (iii) Datasets from multiple parallel and individual studies were tested to evaluate the influence of batch effect. Here, other batches influence such as species or sequencing techniques were also considered. All the datasets were listed in Tab.1. The cell markers of these datasets were collected from the study of the original data [37, 38, 39, 40] or from public libraries such as CellMarker database [36].

Since the strategy of grid search and Louvain were partly introduced in the generation of our initial input, our cmCluster results were firstly compared with the optimized clustering results from these two methods. Besides, a famous consensus clustering method with mathematical approach SC3 will also be taken into consideration. All of the clustering and annotation methods involved in this article could be found in Supplementary Table S6 [35,41, 42, 43].

**Table 1:** Datasets

Datasets	Species	Tissue	Description	Technology	Cell types	Standard labels	Batch	Size (genes*cells)	Reference
10X PBMC	Human	PBMC	Health	10X	Known	Yes	Multi	18,161 *74,287	[37]
GSE136103	Human	Liver	Liver cirrhosis	10X	Unknown	No	Single	23,331 *27,787	[38]
SRP135960	Mouse	Brain	CD‐1 or Wnt1‐Cre:R26RTomato mice	10X	Unknown	Yes	Multi	22,059 *156,864	[39]
GSM2967053	Mouse	Lung	C57BL/6JN mice	Smart‐seq2	Known	Yes	Single	17,396 *1,825	[40]
GSM2967057	Mouse	Pancreas	C57BL/6JN mice	Smart‐seq2	Known	Yes	Single	23434*1960	[40]

## SUPPLEMENTARY MATERIALS

The supplementary materials can be found online with this article at https://doi.org/10.15302/J‐QB‐022‐0311

## COMPLIANCE WITH ETHICS GUIDELINES

The authors Yuwei Huang, Huidan Chang, Xiaoyi Chen, Jiayue Meng, Mengyao Han, Tao Huang, Liyun Yuan and Guoqing Zhang declare that they have no conflicts of interest.

This article does not contain any studies with human or animal subjects performed by any of the authors. All procedures performed in studies were in accordance with the ethical standards of the institutional and/or national research committee and with the 1964 Helsinki declaration and its later amendments or comparable ethical standards.

## Supporting information

Supplementary Information
