# Supplementary material for: A cell marker‐based clustering strategy (cmCluster) for precise cell type identification of scRNA‐seq data
Source: Quant Biol. 2023 Jun 1;11(2):163–74. doi: 10.15302/J-QB-022-0311 (PMC12807422; doi:10.15302/J-QB-022-0311)
Supplement: Supplementary file 1 — Supplementary Information [file QUB2-11-163-s001.pdf]

Supplementary data

The cell line data of Zheng underlying this article is available in 10X Genomics, at https://support.10xgenomics.com/single-cell-gene-expression/datasets. The novel cell type data of Ramachandran underlying this article is available at NCBI as GEO accession GSE136103. The nerve system datasets of Zeisel underlying this article are available at http://mousebrain.org/downloads.html. The lung and pancreas datasets of Nicholas underlying this article are available at https://tabula-muris.ds.czbiohub.org/. The CellMarker database was available at http://bio-bigdata.hrbmu.edu.cn/CellMarker/. Accessed 8 Jan 2019.

All codes for cmCluster are available at https://github.com/huangyuwei301/cmCluster.

Preprocessing and clustering the single cell data

Raw scRNA-seq data needed pretreatment to yield reliable clusters. This strategy started with a filter expression matrix that was filtered and normalized by Seurat [35] after mapping raw fastq reads through Cell Ranger [36]. We used Seurat to sequentially read in count matrices and merged them through RunMultiCCA [18] where genes expressed in fewer than three cells in a sample were dropped. Values were filtered according to the mitochondrial gene expression level and the number of genes expressed in cells. We excluded the 1% upper and lower limit cells through the distribution of the expression level of mitochondrial genes and the number of expression genes, respectively, for simulated data and cell line data. As for the novel cell type data, we filtered the cells with expression genes fewer than 300 or mitochondrial gene content more than 30% of the total Unique Molecular Identifier (UMI) count as described in the description of the original article [37]. Next, we searched for highly variable genes, standardized to obtain the combined expression matrix for all datasets and performed Principal Component Analysis (PCA [38]) to reduce dimension for clustering.

After preprocessing, clustering was performed with the Louvain [21, 35] using three parameters including Principal Component (PC) , K-nearest neighbors (K) and Resolution (R).

Supplementary figures

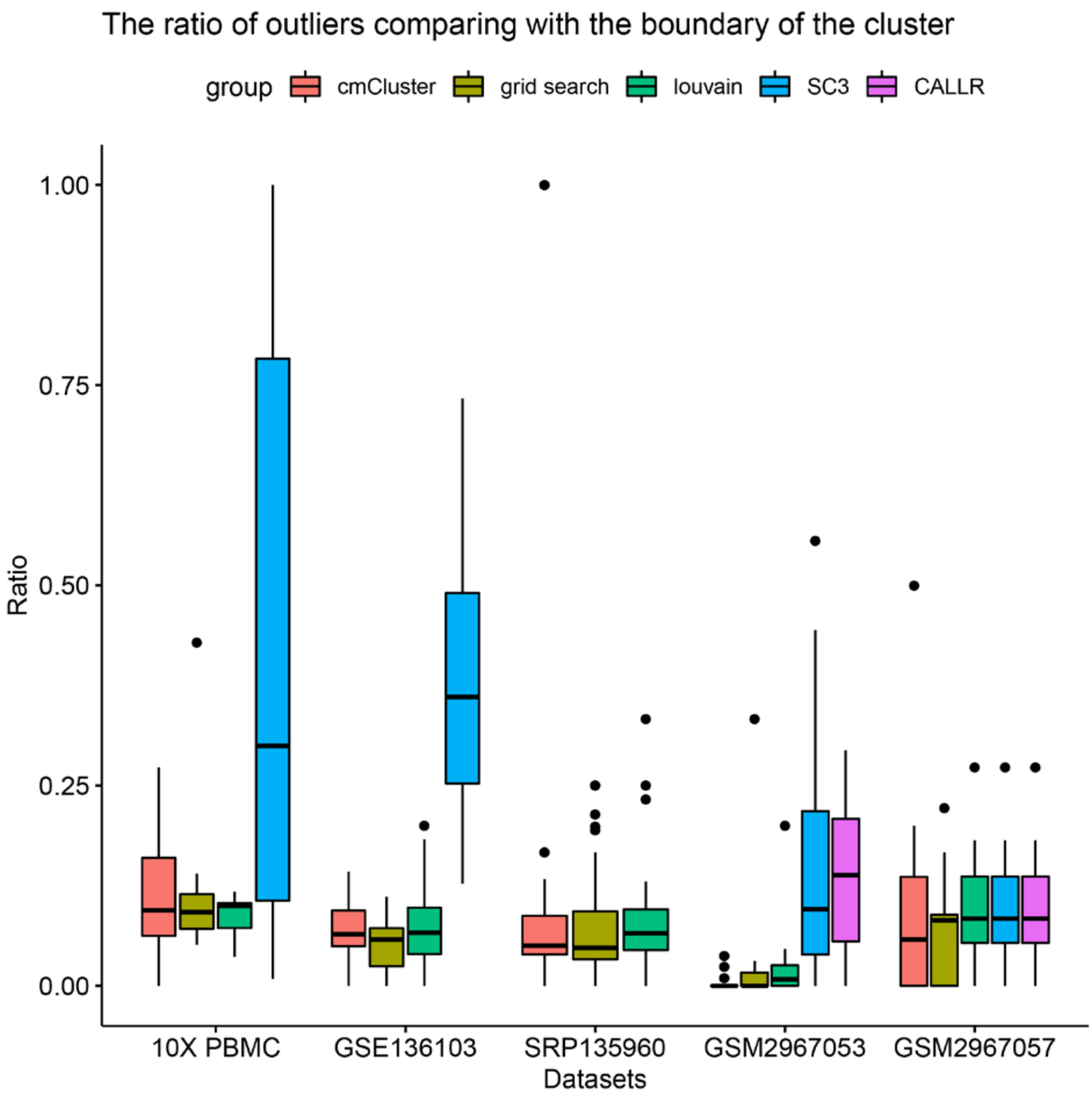

Figure S1. The ratio of outliers comparing with the boundary of cluster in all datasets.

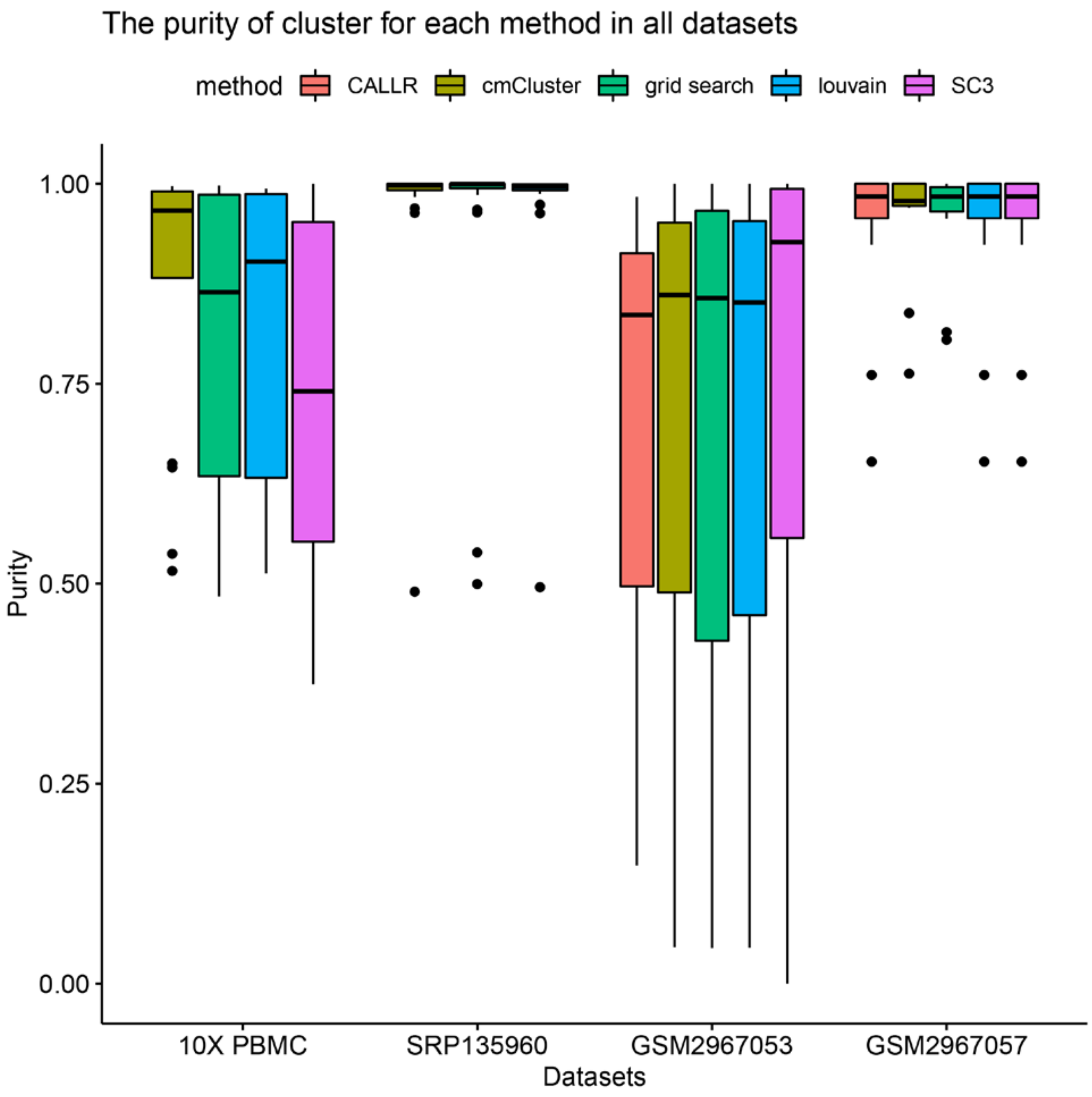

Figure S2. The purity of cluster for all groups in datasets with standard label.

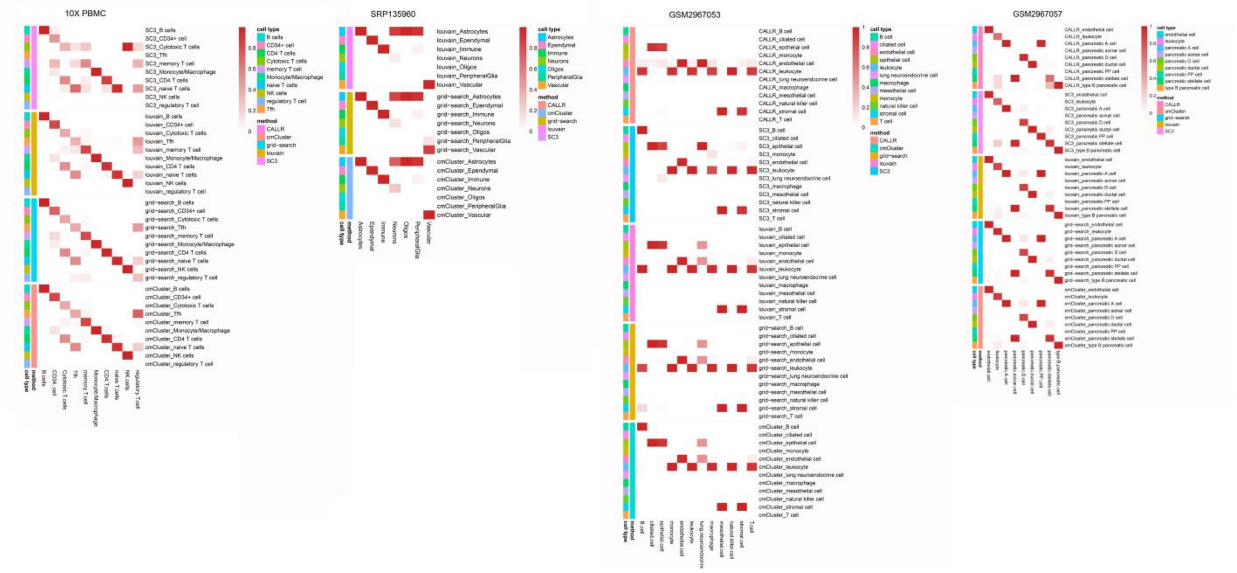

Figure S3. The confusion matrix between predicted and true cell types for cmCluster and other methods in datasets with standard label.

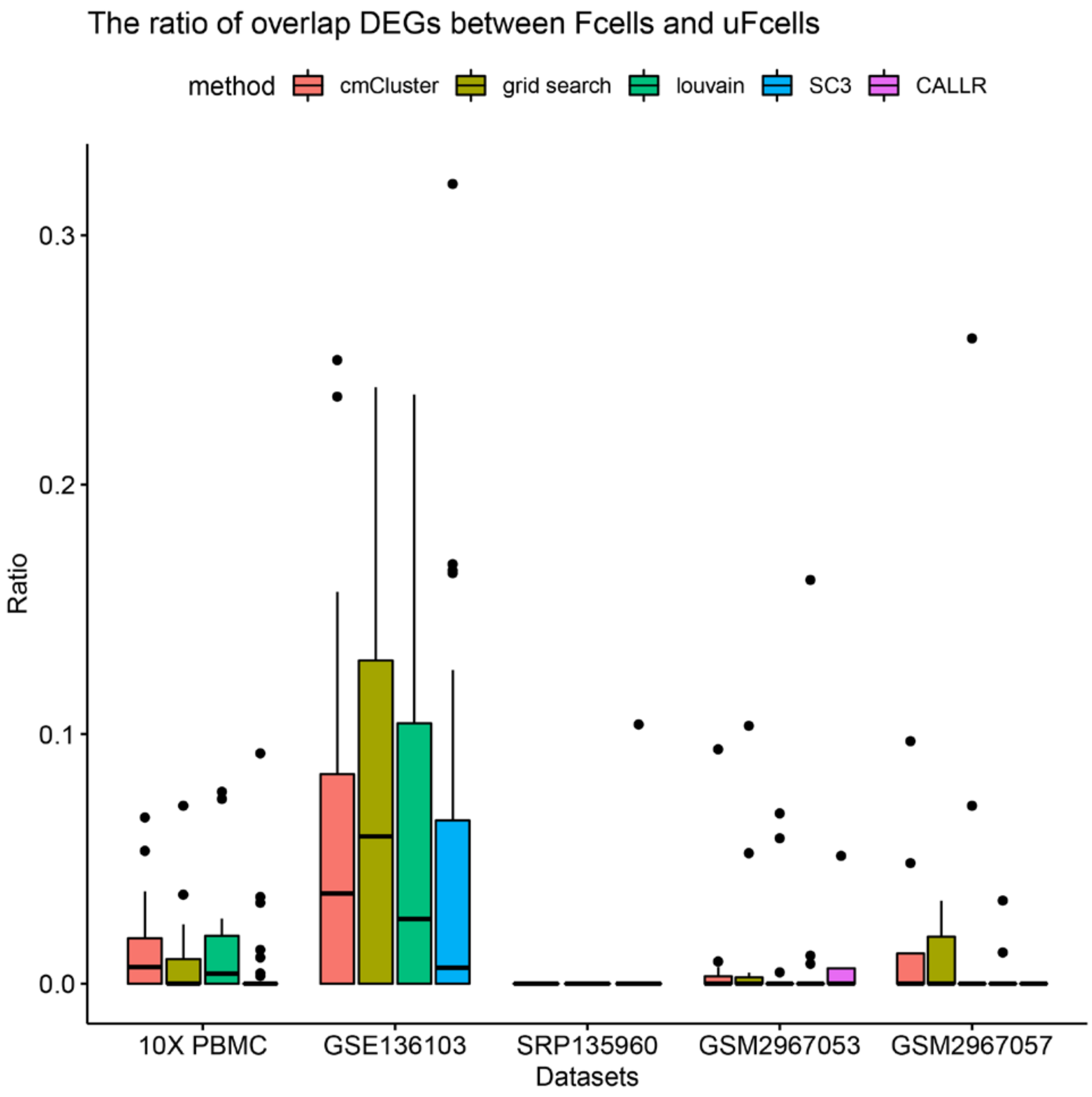

Figure S4. The overlap of DEG between noise and agree cells in all dataset.

Supplementary Table1. gene markers for 10 cell types in 10X PBMC.

Cell Types	Gene Marker
B cells	CD19, CD79A, MS4A1
NK cells	GZMB, FCGR3A, NCAM1
CD4 T cells	S100B,CD8A
Tfh	GATA3,AQP3
Monocyte/Macrophage	CD14,LYZ,CD68
memory T cell	CCR10,LGALS1
regulatory T cell	IL10RA,SIT1
Cytotoxic T cells	CCL5,GZMK
naive T cells	CCR7,LEF1
CD34+ cell	CYTL1,CD34

Supplementary Table2. gene markers for 10 cell types in GSE136103.

Cell Types	Gene Marker
MP	CD68, ITGAM, ITGAX, HLA-DRA, CSF1R, CD14
pDC	LILRA4, CLEC4C, GZMB
ILC	KLRF1, KLRC1, GZMA, GZMB, NKG7
T cell	CD3D, CD3E, CD3G, CD8A
B cell	CD79A, CD79B, CD19, MS4A1
Plasma cell	CD79A, IGHA2
Mast cell	KIT, TPSAB1, TPSB2
Endothelia	PECAM1, CDH5, ICAM2, KDR, ERG
Mesenchyme	PDGFRB, ACTA2, COL1A1, COL1A2, COL3A1, DES, DCN
Hepatocyte	ALB, TF, TTR, HNF4A, CYP2A6

Supplementary Table3. gene markers for 10 cell types in SRP135960.

Cell Types	Gene Marker
Astrocytes	Timp4,Gfap,Slc6a11,Fam107a
Ependymal	Ccdc153,Cfap126,1500015O10Rik
Immune	P2ry12
Neurons	Gm7271,Hand1
Oligos	Opalin, Hapln2,Neu4,Cnksr3
PeripheralGlia	Tax1bp3,Col12a1
Vascular	Higd1b,Ecscr

Supplementary Table4. gene markers for 10 cell types in GSM2967053.

Cell Types	Gene Marker
leukocyte	Ptprc
monocyte	Csf1r,Ccr2,Cd14,Cx3cr1
macrophage	Marco,Mrc1
T cell	Cd3e,Cd8a
B cell	Cd19,Cd79a
natural killer cell	K1rb1c
stromal cell	Col1a1
endothelia cell	Pecam1
mesothelial cell	Wt1
ciliated cell	Foxj1,Scgb3a2,Msln
lung neuroendocrine cell	Pdpn

Supplementary Table5. gene markers for 10 cell types in GSM2967057.

Cell Types	Gene Marker
endothelial cell	Cdh5,Kdr,Pecam1
leukocyte	Ptprc,Mafb
pancreatic A cell	Isl1,Gcg,Chga,Arx,Mafb,Ppy
pancreatic acinar cell	Amy2b,Cpa1
pancreatic D cell	Sst,Neurog3,Pdx1,Ppy,Chga,Isl1,Hhex
pancreatic ductal cell	Hnf1b,Hhex,Krt19,Spp1
pancreatic PP cell	Arx,Chga,Isl1
pancreatic stellate cell	Pdgfrb,Pdgfra
type B pancreatic cell	Chga,Prss53,Pdx1,Nkx6-1,Slc2a2

Supplementary Table6. cell clustering and identification methods for single cell datasets

Methods	Target	Package	Category	Ref.
Grid search	Clustering	-	Optimal solution	[43]
Louvain	Clustering	Seurat or Scanpy	Community detection	[21, 22]
SC3	Clustering	SC3 and scater	Clustering consensus	[23]
Garnett	Annotation	Garnett and monocle	Supervised learning	[41]
CALLR	Annotation	Glmnet	Semi-supervised learning	[42]
scReClassify	Annotation	scReClassify	Semi-supervised learning	[27]
RaceID	Clustering	RaceID	Searching outliers	[28]
SIMLR	Annotation	SIMLR	Custom distance measurement	[29]
CellAssign	Annotation	cellassign	Pprobability model	[33]
